# Imidazolium 4-amino­benzoate

**DOI:** 10.1107/S160053680904625X

**Published:** 2009-11-11

**Authors:** Rodolfo Moreno-Fuquen, Alan R. Kennedy, Denise Gilmour, R. H. De Almeida Santos, Rommel B. Viana

**Affiliations:** a Departamento de Química - Facultad de Ciencias, Universidad del Valle, Apartado 25360, Santiago de Cali, Colombia; b WestCHEM, Department of Pure and Applied Chemistry, University of Strathclyde, 295 Cathedral Street, Glasgow G1 1XL, Scotland; c Instituto de Química de São Carlos, Universidade de São Paulo, USP, São Carlos, SP, Brazil

## Abstract

In the title salt, C_3_H_5_N_2_^+^·C_7_H_6_NO_2_^−^, the carboxyl­ate group of the 4-amino­benzoate anion forms a dihedral angle of 13.23 (17)° with respect to the benzene ring. There are N—H⋯O hydrogen-bonding inter­actions between the anion and cation, and weak inter­molecular C—H⋯O contacts with carboxyl­ate O-atom acceptors of the 4-amino­benzoate anion result in extended three-dimensional *R*_4_^4^(22) and *R*_5_^6^(30) edge-fused rings along the [100], [010] and [001] directions.

## Related literature

For the anti­microbial and anti­protozoal biological activity of imidazole, see: Kopanska *et al.* (2004 ▶); Sondhi *et al.* (2002 ▶). For the biological activity of 4-amino­benzoic acid, see: Lai & Marsh (1967 ▶); Robinson (1966 ▶). For related structures, see: Moreno-Fuquen *et al.* (1996 ▶, 2009 ▶); McMullan *et al.* (1979 ▶). For hydrogen-bond motifs, see: Etter (1990 ▶). For hydrogen bonds, see: Nardelli (1995 ▶).
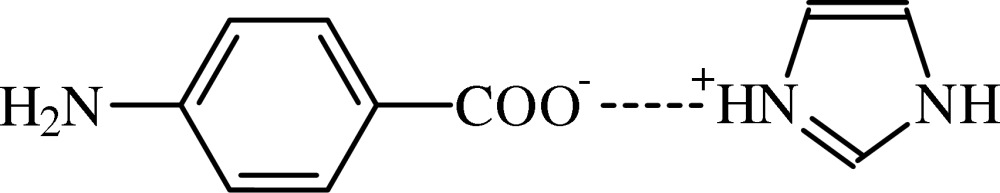


## Experimental

### 

#### Crystal data

C_3_H_5_N_2_^+^·C_7_H_6_NO_2_^−^
*M* *_r_* = 205.22
Monoclinic, 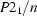

*a* = 7.2038 (5) Å
*b* = 11.6812 (6) Å
*c* = 12.0152 (6) Å
β = 105.223 (6)°
*V* = 975.59 (10) Å^3^
*Z* = 4
Mo *K*α radiation
μ = 0.10 mm^−1^
*T* = 123 K
0.20 × 0.15 × 0.12 mm

#### Data collection

Oxford Diffraction Gemini S diffractometer
Absorption correction: multi-scan (*CrysAlis CCD*; Oxford Diffraction, 2009 ▶) *T*_min_ = 0.945, *T*_max_ = 1.000
8533 measured reflections
2360 independent reflections
1700 reflections with *I* > 2σ(*I*)
*R*_int_ = 0.049

#### Refinement

*R*[*F* ^2^ > 2σ(*F* ^2^)] = 0.048
*wR*(*F* ^2^) = 0.115
*S* = 1.02
2360 reflections
153 parameters
H atoms treated by a mixture of independent and constrained refinement
Δρ_max_ = 0.28 e Å^−3^
Δρ_min_ = −0.36 e Å^−3^

### 

Data collection: *CrysAlis CCD* (Oxford Diffraction, 2009 ▶); cell refinement: *CrysAlis CCD*; data reduction: *CrysAlis RED* (Oxford Diffraction, 2009 ▶); program(s) used to solve structure: *SHELXS97* (Sheldrick, 2008 ▶); program(s) used to refine structure: *SHELXL97* (Sheldrick, 2008 ▶); molecular graphics: *ORTEP-3 for Windows* (Farrugia, 1997 ▶) and *Mercury* (Macrae *et al.*, 2006 ▶); software used to prepare material for publication: *WinGX* (Farrugia, 1999 ▶).

## Supplementary Material

Crystal structure: contains datablocks I, global. DOI: 10.1107/S160053680904625X/fj2253sup1.cif

Structure factors: contains datablocks I. DOI: 10.1107/S160053680904625X/fj2253Isup2.hkl

Additional supplementary materials:  crystallographic information; 3D view; checkCIF report

## Figures and Tables

**Table 1:** Hydrogen-bond geometry (Å, °)

*D*—H⋯*A*	*D*—H	H⋯*A*	*D*⋯*A*	*D*—H⋯*A*
N3—H4*N*⋯O1^i^	0.93 (2)	1.76 (2)	2.6869 (15)	171 (2)
N2—H3*N*⋯O1^ii^	0.956 (19)	1.742 (19)	2.6938 (15)	173.5 (19)
N1—H1*N*⋯O2^iii^	0.94 (2)	2.17 (2)	2.9495 (16)	139.3 (17)
N1—H2*N*⋯O1^ii^	0.89 (2)	2.20 (2)	3.0149 (17)	152.0 (16)
C6—H6⋯O2^iv^	0.95	2.55	3.3533 (16)	142

Symmetry codes: (i) 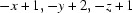; (ii) 
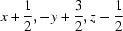
; (iii) 
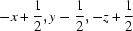
; (iv) 
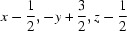
.

**Fig. 1.**
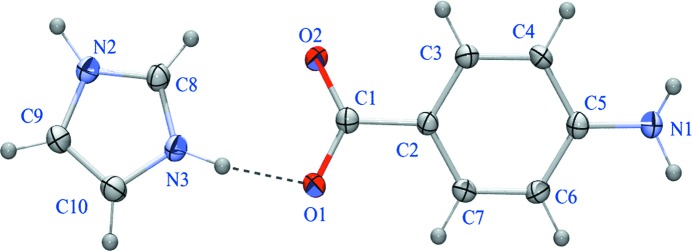
An ORTEP-3 (Farrugia, 1997) plot of the title (I) compound, with the atomic labelling scheme. The shapes of the ellipsoids correspond to 50% probability contours of atomic displacement and, for the sake of clarity, H atoms are shown as spheres of arbitrary radius.

**Fig. 2.**
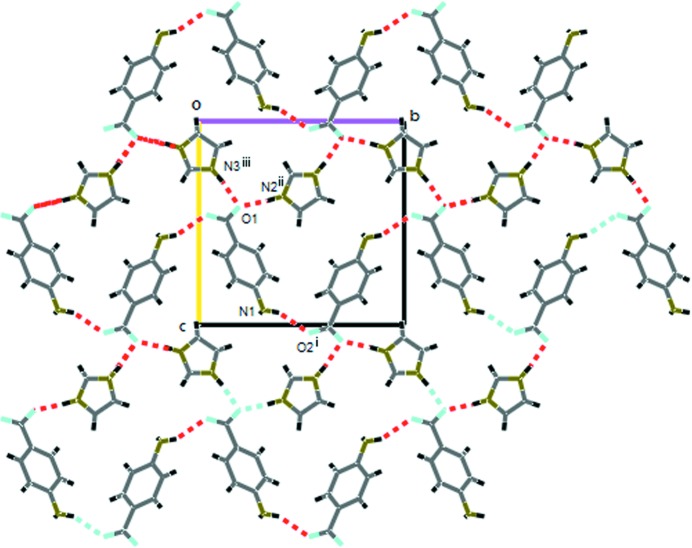
The packing in the unit cell of (I) viewed down the a axis, showing the formation of R56(30) e dge-fused rings and also the hydrogen-bonding interactions as broken lines. Symmetry code: (i) -x + 1/2, y - 1/2, -z + 1/2; (ii) x - 1/2, -y + 3/2, z - 1/2; (iii) -x, -y + 2, -z + 1.

**Fig. 3.**
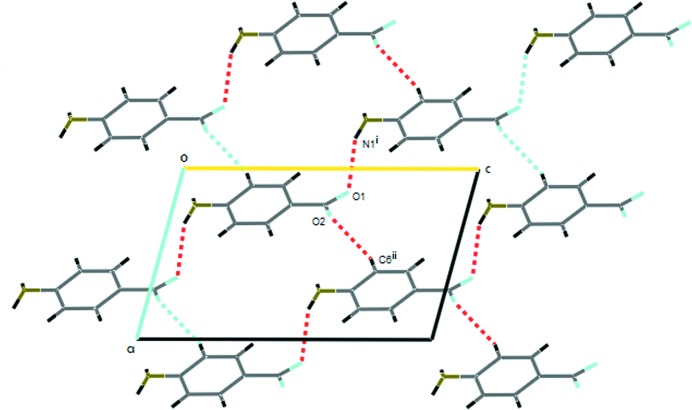
The packing in the unit cell of (I) viewed down the b axis, showing the formation of R44(22) e dge-fused rings and also the hydrogen-bonding interactions as broken lines. Symmetry code: (i) x + 1/2, -y + 3/2, z - 1/2; (ii) x - 1/2, -y + 3/2, z - 1/2.

## supplementary crystallographic information

### Comment

The title adduct, C_7_H_6_NO_2_^-^, C_3_H_5_N_2_^+^ (imidazolium 4-aminobenzoate), (I), is part of a series of studies on imidazole, which have been made in our research group (Moreno-Fuquen *et al.*, 2009). Imidazole derivatives have a wide variety of agents holding biological activities and is used in the field of pharmaceuticals and medicine like antimicrobial and antiprotozoal (Kopanska *et al.*, 2004) or anti-inflammatory agents (Sondhi *et al.*, 2002). In turn, 4-Aminobenzoic acid (PABA) (Lai & Marsh, 1967) is an important biological molecule, involving the synthesis of folic acid (Robinson, 1966) and promoting the extension of hydrogen-bonded network structures. To continue research on the structural behavior of the imidazole molecule with different hydrogen bond donors, the system imidazolium 4-aminobenzoate adduct (I), is reported. The 4-aminobenzoic acid and 4-nitropyridine N-oxide (PABA+NPNO) molecular complex (Moreno-Fuquen *et al.*, 1996) and the PABA and imidazole (IM) free molecules (McMullan *et al.*, 1979) may be used as reference systems in order to compare to the title imidazolium salt. The molecular structure of (I) is shown in Fig. 1. The title compound shows a dihedral angle of 23.71 (8)°, between benzene and imidazole planes. In turn, the carboxylate group of 4-aminobenzoate shows a dihedral angle of 13.23 (17)° with respect to the benzene ring, following the same structural behavior of the group in the free PABA molecule and in the PABA+NPNO adduct. The (PABA), as well as other organic acids, shows the formation of centrosymmetric hydrogen-bonded dimers in its structure. As a product of the reaction with imidazole (IM), the dimer in the PABA molecule is broken, and begins the transference of the proton to the basic N-atom of the IM molecule forming the title adduct. Some structural changes in the formation of the imidazolium salt, are observed: N2—C8 bond length changes from 1.358 in the free IM molecule, to 1.3281 (17) Å in (I); C1—O1 and C6—C7 bond lengths change from 1.210 (4) and 1.366 (5) Å in the (PABA +NPNO) adduct to 1.2368 (16) and 1.3850 (18) Å in the title adduct. The other bond lengths and bond angles of (I) are in good agreement with the standard values and correspond to those observed in the IM free molecule and (PABA+NPNO) reference systems. The formation of the salt, resulting in N—H···O hydrogen-bonding interactions and C—H···O intermolecular weak contacts with carboxylate O-atoms acceptors: The two components of the adduct are connected *via* intermolecular N—H···O hydrogen bonds and C—H···O weak contacts, (Table 1) (Nardelli, 1995) and these interactions define an infinite three dimensional framework. In a first substructure, the strongest hydrogen bonds N—H···O interactions are responsible for crystal growth. Indeed, there are two intermolecular N—H···O hydrogen bond interactions which link one molecule of PABA and 2 molecules of IM. A third N—H···O hydrogen bond links two PABA molecules. All these interactions link the moieties into molecular sheets that extend in the b and c directions forming *R*_5_^6^(30) (Etter, 1990) edge-fused rings (Fig. 2). In a second substructure, the PABA molecules are linked by N—H···O hydrogen-bonding and intermolecular C—H···O weak interactions which form a *R*_4_^4^(22) e dge-fused rings along a and c directions (Fig 3). All of these interactions define the bulk structure of the crystal.

### Experimental

The synthesis of the title compound (I) was carried out by slow evaporation of equimolar quantities of 4-aminobenzoic acid (0.625 g, 0.0046 mol) and imidazole (0.310 g) in 100 ml of a mixture of dry acetonitrile. Colourless blocks of a good quality, suitable for X-ray analysis with a melting point of 371 (1) K were obtained. The initial reagents were purchased from Aldrich Chemical Co., and were used as received.

### Refinement

All H-atoms were located from difference maps and were positioned geometrically and refined using a riding model with C–H= 0.93–0.97 Å and *U*_iso_(H)= 1.2*U*_eq_(C).

### Crystal data

**Table:**

C_3_H_5_N_2_^+^·C_7_H_6_NO_2_^−^	*F*(000) = 432
*M**_r_* = 205.22	*D*_x_ = 1.397 Mg m^−^^3^
Monoclinic, *P*2_1_/*n*	Melting point: 443.0(10) K
Hall symbol: -P 2yn	Mo *K*α radiation, λ = 0.71073 Å
*a* = 7.2038 (5) Å	Cell parameters from 3056 reflections
*b* = 11.6812 (6) Å	θ = 2.5–30.9°
*c* = 12.0152 (6) Å	µ = 0.10 mm^−^^1^
β = 105.223 (6)°	*T* = 123 K
*V* = 975.59 (10) Å^3^	Fragment, colourless
*Z* = 4	0.20 × 0.15 × 0.12 mm

### Data collection

**Table:**

Oxford Diffraction Gemini S diffractometer	2360 independent reflections
Radiation source: fine-focus sealed tube	1700 reflections with *I* > 2σ(*I*)
graphite	*R*_int_ = 0.049
ω scans	θ_max_ = 28.0°, θ_min_ = 2.5°
Absorption correction: multi-scan (*CrysAlis CCD*; Oxford Diffraction, 2009)	*h* = −9→9
*T*_min_ = 0.945, *T*_max_ = 1.000	*k* = −13→15
8533 measured reflections	*l* = −15→15

### Refinement

**Table:**

Refinement on *F*^2^	Secondary atom site location: difference Fourier map
Least-squares matrix: full	Hydrogen site location: inferred from neighbouring sites
*R*[*F*^2^ > 2σ(*F*^2^)] = 0.048	H atoms treated by a mixture of independent and constrained refinement
*wR*(*F*^2^) = 0.115	*w* = 1/[σ^2^(*F*_o_^2^) + (0.067*P*)^2^] where *P* = (*F*_o_^2^ + 2*F*_c_^2^)/3
*S* = 1.02	(Δ/σ)_max_ < 0.001
2360 reflections	Δρ_max_ = 0.28 e Å^−^^3^
153 parameters	Δρ_min_ = −0.36 e Å^−^^3^
0 restraints	Extinction correction: *SHELXL97* (Sheldrick, 2008), Fc^*^=kFc[1+0.001xFc^2^λ^3^/sin(2θ)]^-1/4^
Primary atom site location: structure-invariant direct methods	Extinction coefficient: 0.213 (14)

### Special details

**Table:**

Geometry. All e.s.d.'s (except the e.s.d. in the dihedral angle between two l.s. planes) are estimated using the full covariance matrix. The cell e.s.d.'s are taken into account individually in the estimation of e.s.d.'s in distances, angles and torsion angles; correlations between e.s.d.'s in cell parameters are only used when they are defined by crystal symmetry. An approximate (isotropic) treatment of cell e.s.d.'s is used for estimating e.s.d.'s involving l.s. planes.
Refinement. Refinement of *F*^2^ against ALL reflections. The weighted *R*-factor *wR* and goodness of fit *S* are based on *F*^2^, conventional *R*-factors *R* are based on *F*, with *F* set to zero for negative *F*^2^. The threshold expression of *F*^2^ > σ(*F*^2^) is used only for calculating *R*-factors(gt) *etc*. and is not relevant to the choice of reflections for refinement. *R*-factors based on *F*^2^ are statistically about twice as large as those based on *F*, and *R*- factors based on ALL data will be even larger.

### Fractional atomic coordinates and isotropic or equivalent isotropic displacement parameters (Å^2^)

**Table:**

	*x*	*y*	*z*	*U*_iso_*/*U*_eq_	
O1	0.13700 (14)	0.80374 (8)	0.58020 (7)	0.0227 (3)	
O2	0.27846 (15)	0.96304 (8)	0.54056 (8)	0.0243 (3)	
N1	0.2159 (2)	0.68622 (11)	0.07332 (10)	0.0247 (3)	
N2	0.77001 (18)	0.91059 (11)	0.13156 (10)	0.0246 (3)	
N3	0.84977 (17)	1.06234 (10)	0.23605 (10)	0.0231 (3)	
C1	0.2104 (2)	0.86668 (12)	0.51343 (11)	0.0201 (3)	
C2	0.21134 (19)	0.81695 (11)	0.39852 (10)	0.0187 (3)	
C3	0.3206 (2)	0.86798 (12)	0.33249 (11)	0.0218 (3)	
H3	0.3954	0.9338	0.3613	0.026*	
C4	0.3225 (2)	0.82474 (12)	0.22568 (11)	0.0222 (3)	
H4	0.3975	0.8615	0.1819	0.027*	
C5	0.2148 (2)	0.72728 (12)	0.18135 (11)	0.0206 (3)	
C6	0.1034 (2)	0.67656 (11)	0.24708 (11)	0.0212 (3)	
H6	0.0274	0.6112	0.2181	0.025*	
C7	0.10240 (19)	0.72053 (11)	0.35398 (11)	0.0204 (3)	
H7	0.0265	0.6845	0.3977	0.025*	
C8	0.7922 (2)	0.95506 (12)	0.23596 (12)	0.0238 (3)	
H8	0.7701	0.9158	0.3005	0.029*	
C9	0.8142 (2)	0.99372 (12)	0.06171 (12)	0.0268 (4)	
H9	0.8099	0.9859	−0.0176	0.032*	
C10	0.8650 (2)	1.08847 (13)	0.12684 (12)	0.0266 (4)	
H10	0.9039	1.1597	0.1021	0.032*	
H1N	0.175 (3)	0.6098 (17)	0.0598 (16)	0.048 (5)*	
H2N	0.323 (3)	0.7036 (15)	0.0534 (16)	0.044 (5)*	
H3N	0.729 (3)	0.8341 (16)	0.1097 (16)	0.045 (5)*	
H4N	0.860 (3)	1.1143 (18)	0.2961 (19)	0.060 (6)*	

### Atomic displacement parameters (Å^2^)

**Table:**

	*U*^11^	*U*^22^	*U*^33^	*U*^12^	*U*^13^	*U*^23^
O1	0.0297 (6)	0.0203 (5)	0.0205 (5)	−0.0014 (4)	0.0109 (4)	0.0007 (4)
O2	0.0344 (6)	0.0186 (5)	0.0202 (5)	−0.0044 (4)	0.0076 (4)	−0.0017 (4)
N1	0.0300 (8)	0.0252 (7)	0.0200 (6)	−0.0044 (6)	0.0085 (5)	−0.0036 (5)
N2	0.0290 (7)	0.0209 (6)	0.0257 (6)	−0.0012 (5)	0.0103 (5)	−0.0042 (5)
N3	0.0257 (7)	0.0216 (6)	0.0233 (6)	−0.0008 (5)	0.0083 (5)	−0.0044 (5)
C1	0.0210 (7)	0.0198 (7)	0.0188 (6)	0.0031 (6)	0.0041 (5)	0.0016 (5)
C2	0.0213 (7)	0.0176 (7)	0.0167 (6)	0.0013 (5)	0.0042 (5)	0.0011 (5)
C3	0.0252 (8)	0.0194 (7)	0.0209 (6)	−0.0029 (6)	0.0063 (6)	−0.0002 (5)
C4	0.0260 (8)	0.0225 (7)	0.0199 (6)	−0.0033 (6)	0.0095 (5)	0.0018 (5)
C5	0.0215 (7)	0.0214 (7)	0.0178 (6)	0.0029 (6)	0.0033 (5)	0.0004 (5)
C6	0.0216 (7)	0.0186 (7)	0.0222 (6)	−0.0026 (6)	0.0038 (5)	−0.0010 (5)
C7	0.0208 (7)	0.0197 (7)	0.0215 (7)	−0.0009 (6)	0.0066 (5)	0.0021 (5)
C8	0.0272 (8)	0.0217 (7)	0.0231 (7)	0.0020 (6)	0.0076 (6)	−0.0020 (5)
C9	0.0305 (8)	0.0281 (8)	0.0247 (7)	−0.0031 (6)	0.0122 (6)	−0.0021 (6)
C10	0.0308 (8)	0.0244 (8)	0.0269 (7)	−0.0005 (6)	0.0118 (6)	0.0021 (6)

### Geometric parameters (Å, °)

**Table:**

O1—C1	1.2987 (16)	C2—C7	1.3964 (18)
O2—C1	1.2368 (16)	C3—C4	1.3825 (18)
N1—C5	1.3858 (17)	C3—H3	0.9500
N1—H1N	0.94 (2)	C4—C5	1.4016 (19)
N1—H2N	0.89 (2)	C4—H4	0.9500
N2—C8	1.3281 (17)	C5—C6	1.397 (2)
N2—C9	1.3745 (19)	C6—C7	1.3850 (18)
N2—H3N	0.956 (19)	C6—H6	0.9500
N3—C8	1.3200 (19)	C7—H7	0.9500
N3—C10	1.3794 (18)	C8—H8	0.9500
N3—H4N	0.93 (2)	C9—C10	1.349 (2)
C1—C2	1.4996 (18)	C9—H9	0.9500
C2—C3	1.3900 (19)	C10—H10	0.9500
			
C5—N1—H1N	114.3 (12)	C5—C4—H4	119.7
C5—N1—H2N	113.1 (12)	N1—C5—C6	121.89 (13)
H1N—N1—H2N	115.1 (18)	N1—C5—C4	119.88 (13)
C8—N2—C9	108.10 (12)	C6—C5—C4	118.19 (12)
C8—N2—H3N	125.2 (12)	C7—C6—C5	120.69 (12)
C9—N2—H3N	126.6 (12)	C7—C6—H6	119.7
C8—N3—C10	108.23 (12)	C5—C6—H6	119.7
C8—N3—H4N	125.5 (13)	C6—C7—C2	121.02 (13)
C10—N3—H4N	125.7 (13)	C6—C7—H7	119.5
O2—C1—O1	123.37 (12)	C2—C7—H7	119.5
O2—C1—C2	119.86 (12)	N3—C8—N2	109.39 (13)
O1—C1—C2	116.77 (12)	N3—C8—H8	125.3
C3—C2—C7	118.20 (12)	N2—C8—H8	125.3
C3—C2—C1	119.96 (12)	C10—C9—N2	107.24 (13)
C7—C2—C1	121.83 (12)	C10—C9—H9	126.4
C4—C3—C2	121.20 (13)	N2—C9—H9	126.4
C4—C3—H3	119.4	C9—C10—N3	107.04 (13)
C2—C3—H3	119.4	C9—C10—H10	126.5
C3—C4—C5	120.68 (13)	N3—C10—H10	126.5
C3—C4—H4	119.7		
			
O2—C1—C2—C3	12.6 (2)	C4—C5—C6—C7	−1.1 (2)
O1—C1—C2—C3	−167.05 (12)	C5—C6—C7—C2	0.5 (2)
O2—C1—C2—C7	−166.47 (12)	C3—C2—C7—C6	0.2 (2)
O1—C1—C2—C7	13.89 (18)	C1—C2—C7—C6	179.26 (12)
C7—C2—C3—C4	−0.2 (2)	C10—N3—C8—N2	0.15 (16)
C1—C2—C3—C4	−179.26 (12)	C9—N2—C8—N3	−0.43 (16)
C2—C3—C4—C5	−0.5 (2)	C8—N2—C9—C10	0.55 (17)
C3—C4—C5—N1	178.96 (13)	N2—C9—C10—N3	−0.45 (17)
C3—C4—C5—C6	1.1 (2)	C8—N3—C10—C9	0.19 (16)
N1—C5—C6—C7	−178.90 (13)		

### Hydrogen-bond geometry (Å, °)

**Table:**

*D*—H···*A*	*D*—H	H···*A*	*D*···*A*	*D*—H···*A*
N3—H4N···O1^i^	0.93 (2)	1.76 (2)	2.6869 (15)	171 (2)
N2—H3N···O1^ii^	0.956 (19)	1.742 (19)	2.6938 (15)	173.5 (19)
N1—H1N···O2^iii^	0.94 (2)	2.17 (2)	2.9495 (16)	139.3 (17)
N1—H2N···O1^ii^	0.89 (2)	2.20 (2)	3.0149 (17)	152.0 (16)
C6—H6···O2^iv^	0.95	2.55	3.3533 (16)	142

Symmetry codes: (i) −*x*+1, −*y*+2, −*z*+1; (ii) *x*+1/2, −*y*+3/2, *z*−1/2; (iii) −*x*+1/2, *y*−1/2, −*z*+1/2; (iv) *x*−1/2, −*y*+3/2, *z*−1/2.
